# Nephrolithiasis in elderly population; effect of demographic characteristics

**DOI:** 10.15171/jnp.2017.11

**Published:** 2016-12-17

**Authors:** Emadoddin Moudi, Seyed Reza Hosseini, Ali Bijani

**Affiliations:** ^1^Department of Urology, Shahid Beheshti Hospital, Babol University of Medical Sciences, Babol, Iran; ^2^Social Determinants of Health Research Center, Health Research Institute, Babol University of Medical Sciences, Babol, Iran; ^3^General physician, Social Determinants of Health Research Center, Health Research Institute, Babol University of Medical Sciences, Babol, Iran

**Keywords:** Kidney calculi, Nephrolithiasis, Metabolic syndrome

## Abstract

**Background:**

Kidney stone (nephrolithiasis) is one of the most common diseases. During the past several decades, its prevalence and incidence have increased markedly in elderly population.

**Objectives:**

This study was conducted to evaluate the risk factors for nephrolithiasis in elderly population.

**Patients and Methods:**

This study was based on the Amirkola Health and Ageing Project (AHAP). Elderly people with kidney stones in every size, type and number were considered to be the case group and other subjects without a history of kidney stones served as control group. Demographic and anthropometric data, smoking, diabetes and metabolic syndrome (MetS), calcium (Ca), vitamin D, parathyroid hormone (PTH), uric acid and urine pH were compared in both groups.

**Results:**

In this study, 1390 elderly people with the mean age of 69.37 ± 7.42 years were evaluated which 202 (14.53%) cases had renal stones. The patients with nephrolithiasis were younger (*P* = 0.010) and had higher uric acid and body mass index (BMI) levels (*P* = 0.041 and *P* = 0.006, respectively). Age <75 years, male gender and BMI ≥30 kg/m^2^ had a significant association with stone formation. The prevalence of diabetes, MetS and smoking in the patients with nephrolithiasis was lower than the subjects without it.

**Conclusions:**

This study suggests that male gender, obesity and age <75 years might be independent risk factors for the development of nephrolithiasis. Hence, low animal protein intake and weight reduction should be included as part of the counseling of senior stone-formers.

**Implication for health policy/practice/research/medical education:**

In a study on 1390 elderly people with the mean age of 69.37 ± 7.42 years, 202 (14.53%) cases had renal stones. The patients with nephrolithiasis were younger and had higher uric acid and BMI levels. Age <75 years, male gender and BMI ≥30 kg/m^2^ had a significant association with stone formation. The prevalence of diabetes, MeS and smoking in the patients with nephrolithiasis was lower than the subjects without it. This study suggests that male gender, obesity and age <75 years might be independent risk factors for the development of nephrolithiasis. Hence, low animal protein intake and weight reduction should be included as part of the counseling of senior stone-formers.

## 1. Background

Kidney stone (nephrolithiasis) is one of the most common diseases, affecting nearly one in 13 women and one in seven men (1). It is a highly prevalent disorder with a lifetime incidence of approximately 13% for Caucasian men and 7% for Caucasian women (2). While the increase occurs across all age groups, most of the mentioned rise in prevalence of stone disease is between 60-74 years in both genders so that about 10%-20% of all patients with nephrolithiasis are older than 65 years (3,4).

Older adults have higher rates of morbidity from kidney stones and higher risk of infectious complications and comorbid conditions such as diabetes, which can increase uric acid stone formation (5,6). Also, most of them are taking medications and vitamin supplements which change their metabolic profile and increase their susceptibility for stone formation (1).

The increasing prevalence of stone disease in the elderly population is a concern, as nephrolithiasis has been associated with multiple comorbidities including hypertension, diabetes mellitus and metabolic syndrome (MeS), coronary artery disease, obesity, and excessive meat consumption (7-10).

Obesity, a body mass index (BMI >30 kg/m^2^), affects more than 300 million people round the world. It is believed that overweight, obesity and larger waist circumference are risk factors for calcium oxalate and uric acid renal stone formation (11). In addition, stone formation may be a marker for increased risk of chronic kidney disease and cardiovascular disease, which are particularly important in older ages. In patients over 70 years of age, 26% has evidence of estimated glomerular filtration rate (GFR) <60 mL/min (12). Since nephrolithiasis is considered as a complex disease, identifying the preventable risk factors may help decrease the number of patients suffering from it.

## 2. Objectives

We conducted this study based on a large elderly population to evaluate the risk factors for nephrolithiasis.

## 3. Patients and Methods

### 3.1. Study population

This study was based on the Amirkola Health and Ageing Project (AHAP) which was approved by the ethics committee of Babol University of Medical Sciences (13). The AHAP involved 2234 people aged more than 60 years who lived in Amirkola, a small town in northern Iran near the Caspian Sea. Initially, the people were visited in their homes and the next day, they came to the Social Determinants of Health Research Centre to complete questionnaires and performing physical examinations and fasting blood samples were taken for biochemical assessments. In total, 1616 seniors participated in this study with response rate of 72.3% (1616/2234).

Elderly people with kidney stones in every size, type and number were considered to be the case group and other subjects without a history of kidney stones served as control group. Demographic and anthropometric data, smoking, diabetes and MeS, and some biochemical parameters in serum and urine were compared in two groups and the elderly who had unreliable or incomplete information on some factors were excluded and the survey was conducted based on data from 1390 elderly people.

The physical activity scale for the elderly (PASE) was also completed. Measurement of blood pressure, hip, waist and neck circumference was conducted. In addition, body weight and height were measured on a standard balance scale with an attached ruler and BMI was calculated as weight in kilograms, divided by height in meters squared.

Fasting venous blood specimens were taken for triglyceride (TG), low-density lipoprotein (LDL-C), high-density lipoprotein (HDL-C), calcium (Ca), vitamin D, and parathyroid hormone (PTH). The serum level of uric acid which might affect the formation of nephrolithiasis, was also measured. Urine analysis for obtaining urine pH was performed too. All analysis were performed the same day in the laboratory of Shahid Beheshti hospital of Babol.

MetS was diagnosed according to the National Cholesterol Education Program Adult Treatment Panel III (NCEP ATP III 2005) (14). Of the components of diagnostic criteria for MetS, abdominal obesity was defined as WC >90 cm in men and >80 cm in women according to the World Health Organization Asia-Pacific obesity criteria (15).

### 3.2. Ethical issues

1) The research followed the tenets of the Declaration of Helsinki; 2) written informed consent was obtained, and they were free to leave the study at any time; and 3) the research was approved by the ethical committee of Babol University of Medical Sciences (Grant ‏# 892917).

### 3.3. Statistical analysis

Data was analyzed using SPSS statistical software version 18.00. For qualitative variables, chi- square and Fisher exact tests were used and *t* test was used for comparison of continuous variables between two groups. In addition, for multivariate evaluation, first the association between risk factors or underlying factors with stone formation was assessed individually using logistic regression model and the variables with a *P* value of <0.25 were forced into final logistic regression model and odds ratio and 95% confidence intervals were calculated. In all cases a *P* value <0.05 was considered being statistically significant.

## 4. Results

In this study, 1390 elderly people with the mean age of 69.37 ± 7.42 years were evaluated. Finally, 202 ones (14.53%) with 95% CI: 12.68%-16.39% were diagnosed with kidney stones. The prevalence of kidney stone in men was higher than women (16.8% vs. 11.6%, *P* = 0.007). Also, its prevalence in the elderly between 60-74 years was higher than 75 years or older (15.9% vs. 10.3%, *P* = 0.013).

Compared to the subjects without nephrolithiasis, demographic status, anthropometric and biochemical measurements and other related variables showed that the patients with nephrolithiasis were younger (*P* = 0.010) and had higher uric acid and BMI levels (*P* = 0.041 and *P* = 0.006, respectively) (Table 1). Also, the prevalence of nephrolithiasis in males was significantly higher than females (16.8% vs. 11.6%, *P* = 0.007).

**Table 1:** Comparison of demographic, anthropometric and biochemical indicators, among the elderly with and without kidney stones

**Variable**	**Nephrolithiasis + ** **n=202**	**Nephrolithiasis − ** **n=1188**	**P value**
Age (Mean ± SD)	67.95 ± 6.18	69.20 ± 7.44	0.010
Gender			0.007
Male (%)	131 (16.8)	648 (83.2)	
Female (%)	71 (11.6)	540 (88.4)	
Smoking			0.877
+ (%)	38 (14.2)	229 (85.8)	
‏–(%)	164 (14.6)	959 (85.4)	
Diabetes			0.788
+ (%)	61 (14.2)	370 (85.8)	
‏–(%)	141 (14.7)	818 (85.3)	
MeS			0.163
+ (%)	159 (15.3)	880 (84.7)	
- (%)	43 (12.3)	308 (87.7)	
Ca (mg/dL) (Mean ± SD)	9.26 ± 0.43	9.25 ± 0.47	0.857
Uric Acid (mg/dL) Mean ± SD)	4.98 ± 1.04	4.83 ± 0.93	0.041
PTH (pg/mL) (Mean ± SD)	5.36 ± 0.70	5.41 ± 0.77	0.264
Vitamin D (ng/mL) (Mean ± SD)	33.99 ± 30.34	34.43 ± 33.14	0.860
BMI (kg/m^2^) (Mean ± SD)	28.00 ± 4.38	27.05 ± 4.53	0.006
Physical activity score (Mean ± SD)	111.10 ± 61.89	106.77 ± 61.33	0.354
Urine pH (Mean ± SD)	5.36 ± 0.70	5.41 ± 0.77	0.332

Abbreviations: PTH, parathyroid hormone; MeS, metabolic syndrome

Thirty-eight (14.2%) of the patients with nephrolithiasis and 141 (14.7%) of the control group were current cigarette smokers (*P* = 0.877).

Vitamin D (25-OH-D) levels were also assessed and 38.2% were vitamin D deficient (<20 ng/mL). The mean vitamin D levels in patients with and without nephrolithiasis were 33.99 ± 30.34 and 34.43±33.14 ng/mL, respectively (*P* = 0.860).

Multivariable models were made for all urinary factors and using logistic regression model and forcing variables whose p value were <0.25 for stone formation into each model, and with Backward method, finally, only male gender, age less than 75 years and BMI were significantly associated with urinary stones (Table 2).

**Table 2:** Association between risk factors with kidney stone formation based on logistic regression model in the elderly population

**Variable**	**OR**	**95% CI OR**	**P value**
Male gender	1.80	1.30-2.49	<0.001
Age <75 years	1.59	1.07-2.35	0.022
BMI (kg/m^2)^			
<25	1	-	0.016
25-29.99	1.31	0.91-1.90	0.153
≥30	1.85	1.21-2.81	0.004

The prevalence of MetS in the patients with nephrolithiasis was lower than the subjects without it (15.3% vs. 84.7%, *P* = 0.163).

The mean physical activity score, Ca, PTH, urine pH and the prevalence of diabetes were not different between two groups (Table 1).

## 5. Discussion

Renal stone disease is a multifactorial disorder and constitutional, environmental and genetic factors play a major role in the development of nephrolithiasis (16). The present study assessed the effects of demographics for nephrolithiasis in elderly population. We found that obesity (BMI ≥30 kg/m^2^) is significantly associated with kidney stone formation in elderly population. In the study of Taylor et al, BMI ≥30 kg/m^2^ was associated with the risk of kidney stone formation (17). The greater incidence of kidney stones in obese patients may be due to an increase in uric acid nephrolithiasis (18). Larger body size may lead to increased urinary excretion of calcium, oxalate, and uric acid, thereby increasing the risk for calcium-containing kidney stones (17).

In this study, the prevalence of nephrolithiasis in males was significantly higher than females. Although men are two to three times more likely than women to develop nephrolithiasis, this gender disparity is narrowing (19). Additionally, male predominance is most seen among middle-aged patients, where it is 2.8 times more common than among women, and progressively, with age increase, it declines to 1.6 times after 90 years of age (20). Although not ensure, this gender gap may be because of the protective effects of estrogen. Such an explanation would be better understood by the finding that this gender gap declines in post-menopausal ages (21,22).

The current study found that the prevalence of MetS in patients with nephrolithiasis was insignificantly lower than the subjects without it (15.3% vs. 84.7%). Contrary to our findings, in the study of Kim et al, nephrolithiasis was associated with MetS and the prevalence of MetS in patients with nephrolithiasis was significantly higher (15.9% vs. 11.2%) (23). MetS is linked directly to the formation of nephrolithiasis. Although there have been many studies on the association between MetS and nephrolithiasis (24-26), it is still unclear that MetS is an independent risk factor for nephrolithiasis, and whether each component of MetS is associated with nephrolithiasis. Although our study result was different from others, however, the 15.3% prevalence of nephrolithiasis in patients with MetS was similar to that of other studies. Kidney stones may be a renal manifestation of MetS and its features should be looked for in renal stone formers (27). In patients with MetS, weight loss and exercise reduce abdominal obesity, insulin resistance and the incidence of cardiovascular events and diabetes. These recommendations could offer beneficial therapeutic options for nephrolithiasis complicated by MetS. It is reasonable and justifiable for the urologist, to recommend these lifestyle changes to patients with both conditions (28).

In this study 14.2% of the patients with nephrolithiasis were cigarette smokers and we found no association between cigarette smoking and nephrolithiasis. In the study of Tamadon et al, 26.5% of the patients smoked and smoking significantly increased the risk of nephrolithiasis (OR = 2.06, 95% CI: 1.06-4.01, p=0.034) (29). Cigarette smoking may induce kidney stone by decreasing urinary flow and increasing serum cadmium in healthy subjects (30).

In this study, the mean urine pH was not different between two groups and the mean uric acid was significantly higher in patients with nephrolithiasis. The major determinant in the formation of idiopathic uric acid stones and various types of kidney stones is an abnormally low urinary pH (31). Systemic acid-base status and urine pH enhance the kidneys’ ability to secrete or reabsorb metabolites and solutes that contribute to the risk of stone formation (32). Also, alkaline reduces solubility of calcium phosphate products, whereas acidic urine pH promotes formation of uric acid or cystine-containing stones (33). Uric acid is an independent predictor for progression in IgA nephropathy which is increased by reduction in glomerular filtration rate and predicts the development of renal insufficiency in individuals with normal renal function (34,35).

We did not find any difference in the prevalence of diabetes and the mean vitamin D level between two groups. Diabetes is associated with an elevated incidence of renal stones. Insulin resistance is believed to change renal acid-base metabolism, which reduces urine pH and increase the risk of uric acid stone disease (36). It may also increase the risk of calcium stone formation by reducing urinary citrate excretion. It suggests the need for careful metabolic assessment in calcium stone formers to prevent recurrent stone formation (37). In the study of Letavernier et al accompanied vitamin D and elevated calcium intake had a synergistic effect on kidney stone formation in this rat model. Hence, healthcare professionals should be cautious about the cumulative risk of kidney stone formation in elderly population which are high risk for osteoporosis and both vitamin D supplementation and calcium may be prescribed for them (38).

Logistic regression analysis showed that age less than 75 years and BMI are significantly associated with kidney stones. Elderly patients present without symptoms of pain, and more likely to have atypical symptoms, including fever, pyuria, and diarrhea (39). This may be one reason why they seek pain medication less than younger people and are less likely to receive medication for expulsive therapy (40). Age and gender increase the risk of kidney stone and should be taken into account when evaluating kidney stone formers (41).

## 6. Conclusions

In conclusion this study suggests that male gender, obesity and age <75 years might be independent risk factors for the development of nephrolithiasis. Just as general recommendations for elderly people with nephrolithiasis, low meat consumption and animal protein intake and weight reduction should be included as part of the counseling of senior stone-formers especially in men. Thus, physicians should consider these factors when assessing elderly patients for kidney stone risk.

## Limitations of the study

It was a single-center non-randomized study. All seniors did not respond our call.

## Acknowledgements

We are thankful to Vice-Chancellery of Health for their assistance in conducting the project.

## Authors’ contribution

EM, and SRH conducted the research. AB analyzed the data. EM and SRH prepared the primary draft. EM and AB edited the final draft. All authors signed the manuscript.

## Conflicts of interest

The authors declare no conflict of interest.

## Funding/support

The authors are grateful to the Vice-Chancellery of Research and Technology of Babol University of Medical Sciences for approving the project (Grant # 892917) and providing financial support.
